# Hyperspectral Imaging Combined With Deep Transfer Learning for Rice Disease Detection

**DOI:** 10.3389/fpls.2021.693521

**Published:** 2021-09-29

**Authors:** Lei Feng, Baohua Wu, Yong He, Chu Zhang

**Affiliations:** ^1^College of Biosystems Engineering and Food Science, Zhejiang University, Hangzhou, China; ^2^Key Laboratory of Spectroscopy Sensing, Ministry of Agriculture and Rural Affairs, Hangzhou, China; ^3^School of Information Engineering, Huzhou University, Huzhou, China

**Keywords:** rice disease detection, hyperspectral imaging, fine-tuning, deep CORAL, deep domain confusion, saliency map

## Abstract

Various rice diseases threaten the growth of rice. It is of great importance to achieve the rapid and accurate detection of rice diseases for precise disease prevention and control. Hyperspectral imaging (HSI) was performed to detect rice leaf diseases in four different varieties of rice. Considering that it costs much time and energy to develop a classifier for each variety of rice, deep transfer learning was firstly introduced to rice disease detection across different rice varieties. Three deep transfer learning methods were adapted for 12 transfer tasks, namely, fine-tuning, deep CORrelation ALignment (CORAL), and deep domain confusion (DDC). A self-designed convolutional neural network (CNN) was set as the basic network of the deep transfer learning methods. Fine-tuning achieved the best transferable performance with an accuracy of over 88% for the test set of the target domain in the majority of transfer tasks. Deep CORAL obtained an accuracy of over 80% in four of all the transfer tasks, which was superior to that of DDC. A multi-task transfer strategy has been explored with good results, indicating the potential of both pair-wise, and multi-task transfers. A saliency map was used for the visualization of the key wavelength range captured by CNN with and without transfer learning. The results indicated that the wavelength range with and without transfer learning was overlapped to some extent. Overall, the results suggested that deep transfer learning methods could perform rice disease detection across different rice varieties. Hyperspectral imaging, in combination with the deep transfer learning method, is a promising possibility for the efficient and cost-saving field detection of rice diseases among different rice varieties.

## Introduction

Rice is one of the most vital crops for human beings and is critical for maintaining food supply. However, the growth of rice is subjected to stresses that are biological and abiotic in nature. Diseases are one of the major threats to rice, causing severe losses in quality and yield (Yang et al., 2019). There are various diseases threatening rice growth, such as bacterial leaf blight, blast, and sheath blight, which are the three major diseases of rice (Kumar et al., 2020; Molla et al., 2020). After being infected with these different diseases, the change in the inner chemical composition of rice varies from variety to variety, with external symptoms of rice leaf also varying.

To ensure the growth of rice, it is crucial to develop rapid and accurate detection methods. For the past decades, numerous rice disease detection methods have been proposed based on the inner and external changes in infected rice leaves (Awaludin et al., 2020; Lin et al., 2020; Shrivastava and Pradhan, 2020). Hyperspectral imaging (HSI) is one of the most studied detection approaches and is sensitive enough to capture the inner chemical difference between a healthy plant and an infected one (Abdulridha et al., 2020; Gao et al., 2020). Therefore, HSI was widely performed for rice disease detection at an early stage (Liu et al., 2018b; Zhu et al., 2019; Conrad et al., 2020).

However, there are still some problems in rice disease detection by HSI. Generally, the dataset of diseased samples is relatively small, which restricts the abilities of machine learning and deep learning methods (Zhao et al., 2018). Besides, the variation in rice varieties makes it challenging to develop a general model for disease detection. Commonly, a model established based on a variety of plants fails to obtain satisfactory results in another variety. In general, an individual model is constructed for each variety of a plant. In addition to the variation in variety, it is also hard to apply a model constructed with plants from a particular period or environmental condition to the same plants from another growth period or environmental condition (Conrad et al., 2020). The reason could be that spectral data of rice from different varieties or different growth periods have different feature spaces and data distributions. Besides, a model developed with data collected from a particular piece of equipment usually fail when applied to another because of differences in absorbance and wavelength shifts (Chen et al., 2020). Several methods were proposed to solve the problem, such as calibration transfer (Li et al., 2015; Liu et al., 2018a; Chen et al., 2020). Yan et al. applied a model built with a spectrometer to predict spectra collected from another with a method based on independent component analysis (Liu et al., 2018a). However, most research studies on calibration transfer methods have a requirement of a certain number of standard samples. Generally, the performance of a calibration transfer has a positive correlation with the number of standard samples (Chen et al., 2020). Overall, the problem of different data distribution (such as variety variation and equipment variation) has restricted the development of real-world applications.

In computer vision, distribution change or domain shift always exists because of many factors such as illumination, pose, background, and image quality (Gong et al., 2012). Recently, transfer learning methods have been used to solve this different kind of data distribution problem, which has been successfully applied to image classification, object detection, face recognition, semantic segmentation, etc. (Wang and Deng, 2018). Transfer learning (TL) is a method that tries to transfer the knowledge learned from one domain (called the source domain) to another different but related domain (called the target domain) (Yang, 2010). In general, transfer learning approaches can be categorized into four classes: instance-based TL, feature-based TL, parameter-based TL, and relational knowledge TL (Pan and Yang, 2010). The conception of these four kinds of TL was discussed in detail in a study by Pan (Pan and Yang, 2010). From another perspective, TL methods could be categorized into traditional and deep-learning-based methods (Tan et al., 2018; Wang and Deng, 2018). Wang et al. reviewed deep-learning-based TL methods in recent years (Wang and Deng, 2018). Many methods were proposed and tested on several standard domain adaption benchmarks in the field of computer vision, such as Office-31 (Saenko et al., 2010; Mingsheng et al., 2015) and Office-Caltech 10 (Gong et al., 2012; Mingsheng et al., 2015). Office-31 is the most used benchmark for transfer learning and consists of 4,652 images within 31 categories collected from three distinct backgrounds: Amazon (A), Webcam (W), and DLSR (D).

Several groups of researchers have introduced these TL methods into hyperspectral classification. Qiu et al. utilized two traditional transfer learning methods for detecting plastic pollution levels in different soil regions with a near-IR sensor (Qiu et al., 2020). Zhao et al. developed a multi-source deep transfer learning framework to classify hyperspectral images within the datasets of Indian Pines and Botswana (Zhao et al., 2020). Jiang et al. obtained satisfactory segmentation results of the hyperspectral image by combining TL and Markov random fields (Jiang et al., 2020). Compared with hyperspectral image classification, there were relatively fewer works focusing on classification based on spectra. In this study, the feasibility of deep transfer learning was investigated for rice disease classification with spectral data. Three deep transfer learning methods, namely, fine-tuning, deep CORrelation ALignment (deep CORAL) (Sun and Saenko, 2016), and deep domain confusion (DDC) (Tzeng et al., 2014), were introduced to address the problem stemming from the domain shift existing in different varieties of rice. The main contents of this survey are as follows: (1) a common CNN architecture was proposed and, respectively, trained with four different varieties of rice for disease-stressed rice classification; (2) the CNN trained on a specific variety of rice was directly applied to another three varieties of rice without fine-tuning (non-fine-tuning) and with fine-tuning; (3) since there are four domains (varieties of rice), deep CORAL methods were applied to all 12 shifts, taking one variety as the source domain and another variety as the target domain; (4) the DDC was applied to all 12 shifts and the performances of all deep transfer learning methods were compared; (5) the saliency map was used to intuitively visualize the key wavelength range captured by the CNN with and without transfer learning.

## Materials and Methods

### Sample Preparation

To verify the effectiveness of the deep transfer learning methods among rice varieties, four varieties of rice were used in this study, namely, Zhongzheyou1 (which was provided by the China Rice Research Institute and Zhejiang Bedoknon Seed Co.), Jiuyou418 (which was selected and bred by the Northern Hybrid Japonica Rice Engineering Technology Center and Xuzhou Institute of Agricultural Science in Xuzhou, Jiangsu Province), Zhongzao39 (which was provided by the China Rice Research Institute), and Xiushui134 (which was jointly selected by the Jiaxing Academy of Agricultural Sciences, Yuyao Seed Management Station, Institute of Genetics and Developmental Biology, Chinese Academy of Sciences, and Zhejiang Jiaxing Crop High-Tech Breeding Center). The four rice varieties are recorded as 01, 02, 03, and 04 for brevity, as shown in Table 1. A month after sowing the seeds into seed plots, the seedlings were transplanted into a laboratory greenhouse and fertilized and watered regularly.

**Table 1:** Rice varieties and their corresponding labels and sample numbers.

**Rice variety**	**Zhongzheyou1**	**Jiuyou418**	**Zhongzao39**	**Xiushui134**
Encoding	01^a^	02	03	04
Total numbers	189	204	228	234
The training set	158	171	191	196
The validation set	16	17	19	19
The test set	15	16	18	19
CK^b^	45	51	60	54
RLB^c^	42	54	51	75
RB^d^	42	42	51	45
RSB^e^	60	57	66	60

*^a^01 represents the rice variety Zhongzheyou1; 02 represents the rice variety Jiuyou418; 03 represents the rice variety Zhongzao39; 04 represents the rice variety Xiushui134.*

*^b^CK represents total healthy samples.*

*^c^RLB represents total samples inoculated by rice leaf blight.*

*^d^RB represents total samples inoculated by rice blast.*

*^e^RSB represents total samples inoculated by rice sheath blast*.

To obtain inoculated samples, an *in vitro* inoculation method was applied. Rice blast and rice sheath blight are fungal diseases, while rice leaf blight is a bacterial disease. Therefore, the fungi of rice blast and rice sheath blight were cultured in a potato dextrose agar medium, and the bacteria of rice blight were cultured in conical flasks.

Healthy rice seedlings of the four varieties with similar growth were selected, washed, and transferred to sterilized plastic flat plates for disease inoculation. Approximately 20 plants from each variety of rice in the three- to five-leaf stage are used, and two to four leaves with visible symptoms per plant were used for analyses. To prevent the plants from drying, the roots were covered with distilled water-sterilized wipes. For rice blast and rice sheath blight inoculation, mycelial pellets were placed on the upper, middle, and lower parts of the leaves, with about three pellets per leaf. For rice leaf blight inoculation, a solution of bacteria was sprayed onto the leaf surface. After inoculation, the plates were covered with plastic wrap and then placed in a room with a temperature of about 26–28°C and a relative humidity of ~80%, and healthy leaves were used as control. Leaves with visible symptoms were collected 4 days later.

Infected leaves cut from the plants were collected for hyperspectral image acquisition. The number of leaves used in this study is presented in Table 1. Three leaves were acquired in an image. In this study, the category value of the healthy samples (CK) was assigned as 0, and the category values of the samples inoculated with rice leaf blight (RLB), rice blast (RB), and rice sheath blight (RSB) were assigned as 1, 2, and 3, respectively. The representative images of healthy and disease samples are shown in Supplementary Figure 1. Regarding data splitting, the samples of each category were. randomly selected into the training set, the validation set and the testing set in a 10:1:1 ratio The number of plants from each rice variety in each category is listed in Table 1.

### Hyperspectral Image Acquisition and Spectra Extraction

A visible/near-IR hyperspectral imaging system covering the spectral range of 379–1,024 nm was used to acquire hyperspectral images of healthy and infected leaves. The hyperspectral imaging system (as shown in Supplementary Figure 2) is formed by an imaging spectrograph (ImSpector V10E; Spectral Imaging Ltd., Oulu, Finland), a highly sensitive 8484-05G CCD camera (Hamamatsu, Hamamatsu City, Japan), and a long camera lens (OLES23; Specim, Spectral Imaging Ltd., Oulu, Finland). The illumination of the system is provided by 150-W tungsten halogen lamps (2,900 Lightsource; Illumination Technologies Inc., Liverpool, NY, United States). This hyperspectral imaging system conducts line scanning, and a moving plate driven by a stepper motor (Isuzu Optics Corp., Taiwan, China) is used to move the samples.

To acquire clear and non-deformable images, the distance between the camera lens and the moving plate, the exposure time of the camera, and the moving speed of the moving plate were adjusted to 13.7 cm, 0.17 s, and 0.7 ms/s, respectively. The acquired hyperspectral images were then corrected using the white reference image (acquired using a piece of pure white Teflon board with nearly 100% reflectance) and the dark reference image (acquired by covering the lens with a black lens cap with nearly 0% reflectance) according to the following equation:


(1) $$\begin{array}{l} I_{C}=\frac{I_{R}-I_{D}}{I_{W}-I_{D}} \end{array}$$

where *I*_*C*_ is the corrected image, *I*_*R*_ is the raw image, *I*_*W*_ is the white reference image, and *I*_*D*_ is the dark reference image.

After image correction, each leaf was defined as a region of interest (ROI), and a wavelet transform (wavelet function: Daubechies 10; decomposition level: 3) was used to de-noise the pixel-wise spectra. The average spectrum of each leaf was calculated as a sample spectrum. The head and the tail of the spectra contained obvious noises and were then dropped, and the full spectra in the range of 448–947 nm (393 wavelengths in total) were used for analysis.

### Model Establishment, Evaluation, and Software

**Figure 1:**
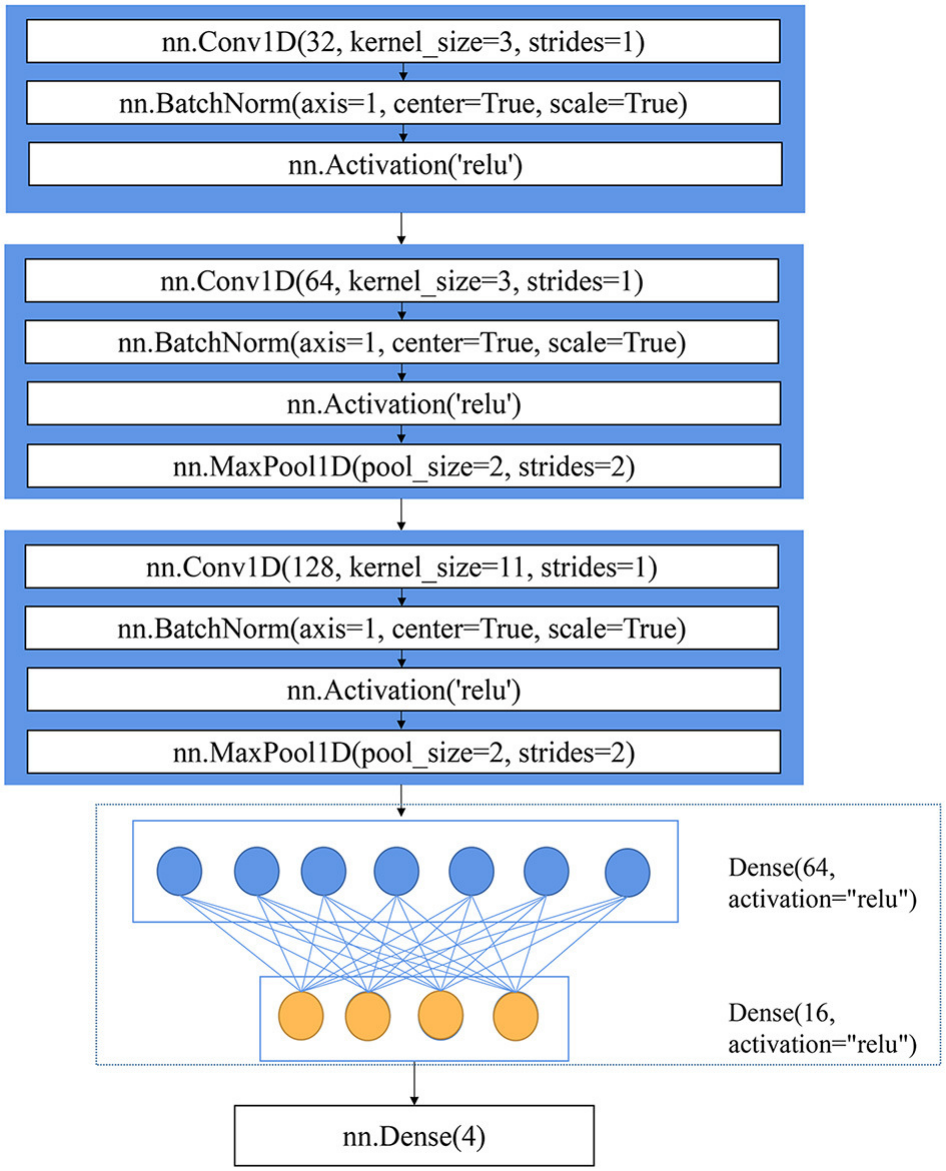
The architecture of CNN.

A self-designed CNN architecture was developed for the classification task, as shown in Figure 1. The CNN consisted of three convolution layers, two fully connected layers, and an output layer. The structure (number of convolution layers/fully connected layers and kernel size) of the CNN in this manuscript was designed using a trial-and-error method. This one-dimensional CNN with different kernels performs convolution operations on the input data, thereby obtaining the global features of the data, while the pooling operation contributed to down-sampling the extracted features and reducing the amount of calculation (Zhong et al., 2021). The CNN could obtain different levels of features because of its stacked convolution layers. If the stride is less than or equal to the size of the convolution kernel, all spectral variables will participate in the convolution operation without losing some information. The stride was set to 1 so that the size of the output feature map of each convolution layer was consistent with the spectral variables. Spectral variables have rich information, and the extracted features are extremely subtle. The shallow semantics generated by the first two convolution layers should retain enough details, and the size of the convolution kernel should be appropriately small and be selected as 3. A large convolution kernel can retain more information in down-sampling. After down-sampling, the convolution kernel size increment was set to 11 (Cai et al., 2018). With the deepening of the CNN, the possibility of feature permutations and combinations increases, that is, the description of each key attribute should be more specific. Therefore, the number of channels should be increased to make the CNN more expressive and cover as many key attributes as possible. In this manuscript, the number of channels continued to double. The rectified linear unit (ReLU) was used as the activation function. The output of the CNN was followed by the softmax function to obtain the probabilities assigned to each class. Cross-entropy loss was used for the classification task. The function of “SoftmaxCrossEntropyLoss” provided by MXNET was used for the softmax operation and loss calculation. The last output layer of the CNN gave a four-value matrix for each sample; then, the prediction category (0, 1, 2, or 3, representing CK, RLB, RB, and RSB, respectively) was obtained according to the four values.

Before being fed into the CNN, the full spectra (393 wavelengths in total) of each class were further implemented with the standardization process. This standardization preprocessing method standardized each sample of the training set by removing the mean and scaling to unit variance, with the same standardization being performed on the test set by a utility class scale in scikit-learn. After standardization preprocessing, the shape of data was (number of samples, number of wavelengths). In order to feed into CNN, the shape of data needs to be reshaped to be (number of samples, 1, number of wavelengths).

To conduct fine-tuning, the CNN was trained on each variety of rice to obtain a decent discriminative performance. Next, the pre-trained parameters of the first three convolution blocks were fronzen and transferred to the CNN to predict another varietry of rice. Fine-tuning assumes that the pre-trained parameters contain the knowledge learned from the source domain and that this knowledge will be equally applicable to the target domain. Since there were four rice varieties, we conducted experiments on all the 12 shifts: 01 → 02, 01 → 03, 01 → 04, 02 → 01, 02 → 03, 02 → 04, 03 → 01, 03 → 02, 03 → 04, 04 → 01, 04 → 02, and 04 → 03.

The spectra extraction of HSI was conducted on Matlab R2019b (MathWorks, Natick, MA, United States). To evaluate the performance of the model, classification accuracy was used, which was the ratio of the correctly classified number of samples to the total sample number. Deep learning was conducted using Python 3 with an MXNET framework (Amazon; Seattle, WA, United States) with GPU acceleration. A computer with an Intel core-i7 8700k CPU, 16 GB of RAM, an NVidia GeForce a GTX1660 GPU (8GB RAM, CUDA cores 1408, CUDA version 9.2.148), and a 256 GB SSD was used for calculation.

### Transfer Learning Methods

#### Fine-Tuning

**Figure 2:**
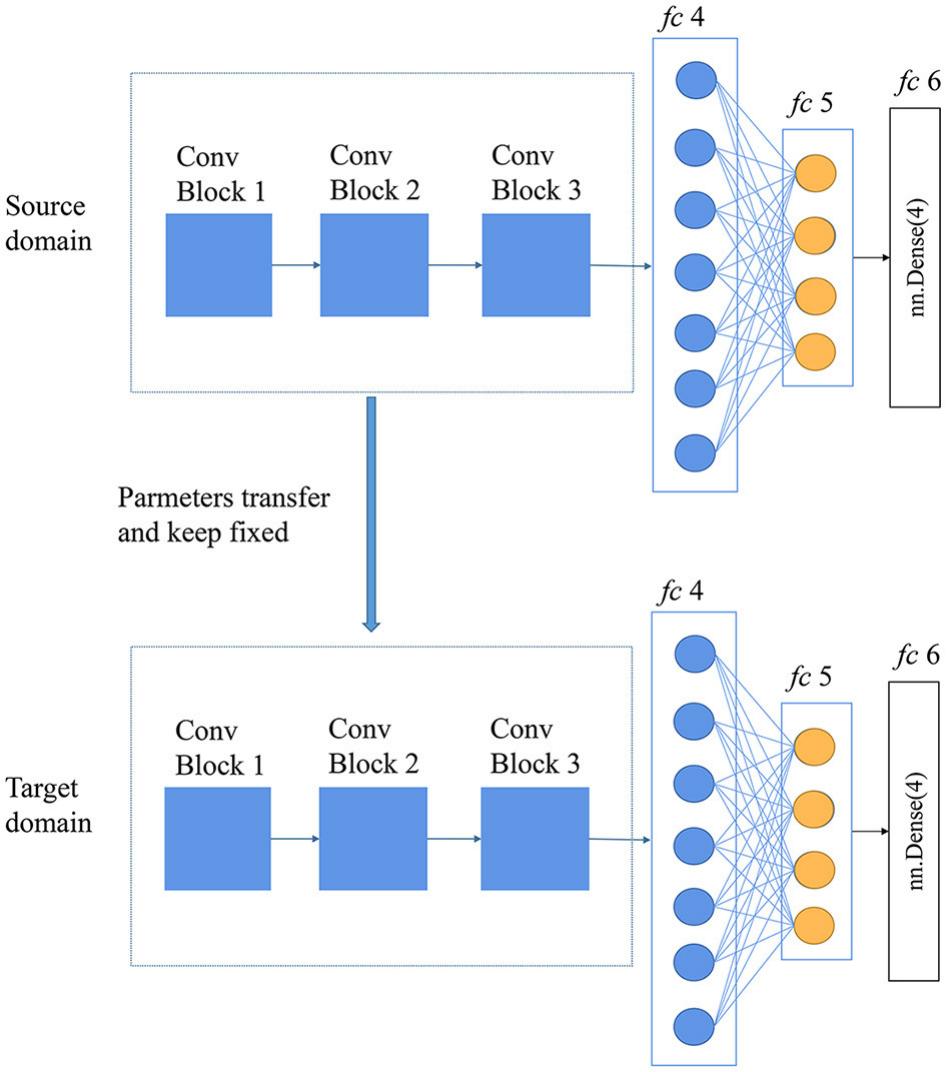
Diagram of the fine-tuning method.

Fine-tuning is a common technique in transfer learning that is widely used in computer vision and natural language processes. It can migrate the knowledge of a pre-trained network based on the source dataset to the target dataset (Oquab et al., 2014). Considerable publications underline the benefits of pre-training deep networks on large datasets (Käding et al., 2016; Zhang et al., 2019). To conduct fine-tuning, the network is first trained on the source dataset, and then, pre-trained parameters are transferred to the target task and kept fixed, with only a few layers (commonly the last few layers) trained on the target dataset (Oquab et al., 2014). In this study, the number of the frozen layers was determined using the “trial and error” method. All layers of the pre-trained CNN are frozen, except for the last three fully connected layers, as shown in Figure 2.

#### Deep CORAL

CORrelation Alignment is a feature-based transfer learning method proposed by Sun et al. (2016) that minimizes the distance between the source domain and the target domain by aligning the second-order statics of source and target distributions (Sun et al., 2016). Furthermore, Sun and Saenko (2016) incorporated CORAL into deep neural networks by constructing a loss function that minimized the difference between the source and target correlations, which is named CORAL loss. CORAL loss is defined as the distance between the second-order statistics (covariances) of the source and target features, the crucial formula of which is as follows (Sun and Saenko, 2016):


(2) $$\begin{array}{l} l_{\text{CORAL}}=\frac{1}{4{\text{d}}^{2}}||C_{S}-C_{T}|{|}_{F}^{2} \end{array}$$

where $||•|{|}_{F}^{2}$ represents the squared matrix Frobenius norm; *C*_*S*_ and *C*_*T*_ represent the feature covariance matrices of the source domain and the target domain, respectively; *d* is the feature dimension in a specific layer in a neural network.

**Figure 3:**
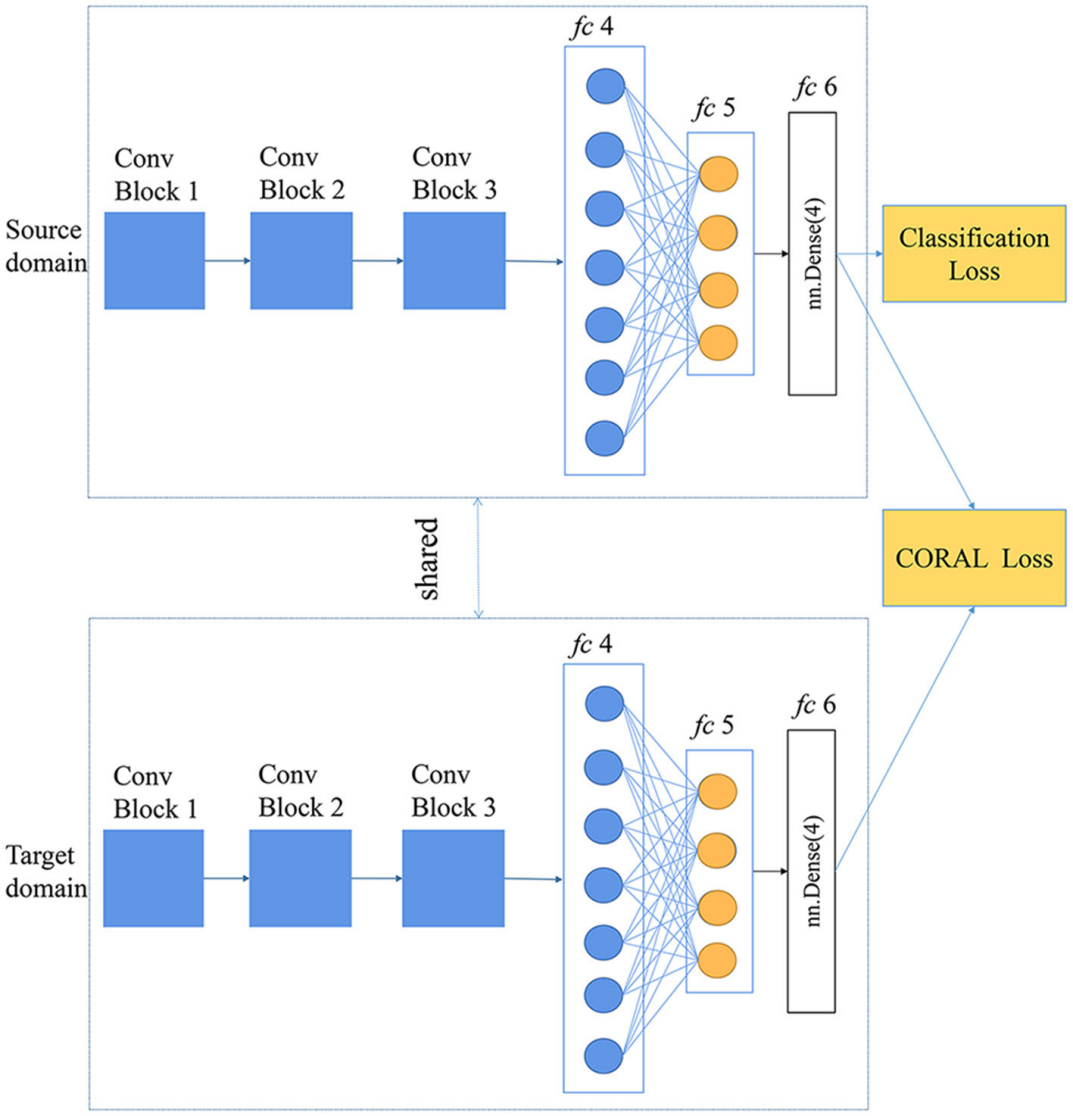
Deep CORAL architecture based on a self-designed CNN.

For generalization and simplicity, in this study, we apply CORAL loss to the *fc6* layer of the self-designed CNN, shown in Figure 3. In the training phase, the batch size was set as 40, and the base learning rate was 0.0001. The weight of the CORAL loss (λ) was set to 0.01 at the initial stage and then expanded to 0.1 and 1. Adding CORAL loss was of help to learn the feature representation that was discriminative and that minimized the distance between the source and the target domain.

#### Deep Domain Confusion

Directly training a classifier using only the source data often leads to inferior performance in the target domain (Tzeng et al., 2014). Deep domain confusion uses domain confusion loss based on the maximum mean discrepancy (MMD) to automatically learn a feature representation jointly trained to optimize for classification and domain invariance (Tzeng et al., 2014). Maximum mean discrepancy is a standard distribution distance between the embeddings of the probability distributions in a reproducing kernel Hilbert space (Gretton et al., 2012; Ghifary et al., 2014). The difference and relationship between CORAL and MMD were discussed in this study (Sun et al., 2016). The key point of DDC is to learn the feature representation that minimizes the distance between domains and is conducive to training a strong classifier at the same time. The approach to meet both these criterias (minimizing distance between domains and training a strong classifier) is to minimize the loss:


(3) $$\begin{array}{l} L=L_{C}\left(X_{S},y\right)+\lambda MMD^{2}\left(X_{S},X_{T}\right) \end{array}$$

where *L*_*C*_(*X*_*S*_, *y*) represents classification loss on the source domain and *MMD*^2^(*X*_*S*_, *X*_*T*_) represents the distance between the source data, *X*_*S*_, and the target data, *X*_*T*_. The hyperparameter λ determines the weight of distance loss.

**Figure 4:**
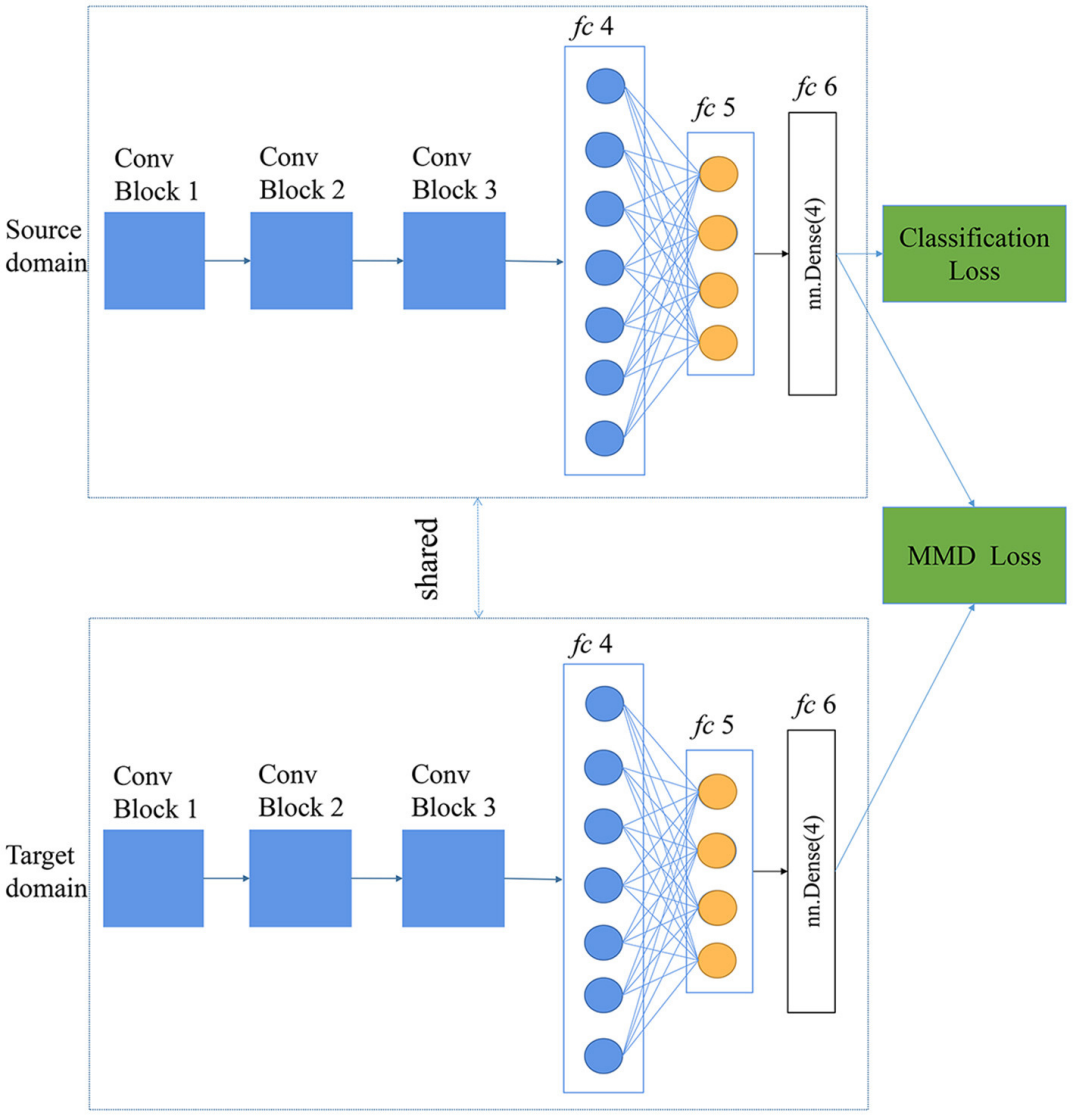
Deep Domain Confusion (DDC) architecture based on a self-designed CNN.

In this study, the same CNN architecture was used in deep CORAL and DDC. Maximum mean discrepancy loss was calculated with the features of the last layer (*fc*6), which is shown in Figure 4. In the training phase, the batch size was set to 40, the base learning rate was 0.0001, and the weight of the MMD loss (λ) was set to 0.001 at the initial stage. Adding MMD loss into total loss was of help to learning the feature representation that was discriminative and minimized the distance between the source and the target domain.

Furthermore, the relationship between epochs and the accuracy of the training set of the target domain with deep CORAL and DDC are provided in Supplementary Figure 3, which illustrates the change in training accuracy and train loss as the change in epochs. The training process took about 286.85 s per 1,000 epochs.

## Results

### Spectral Profile

**Figure 5:**
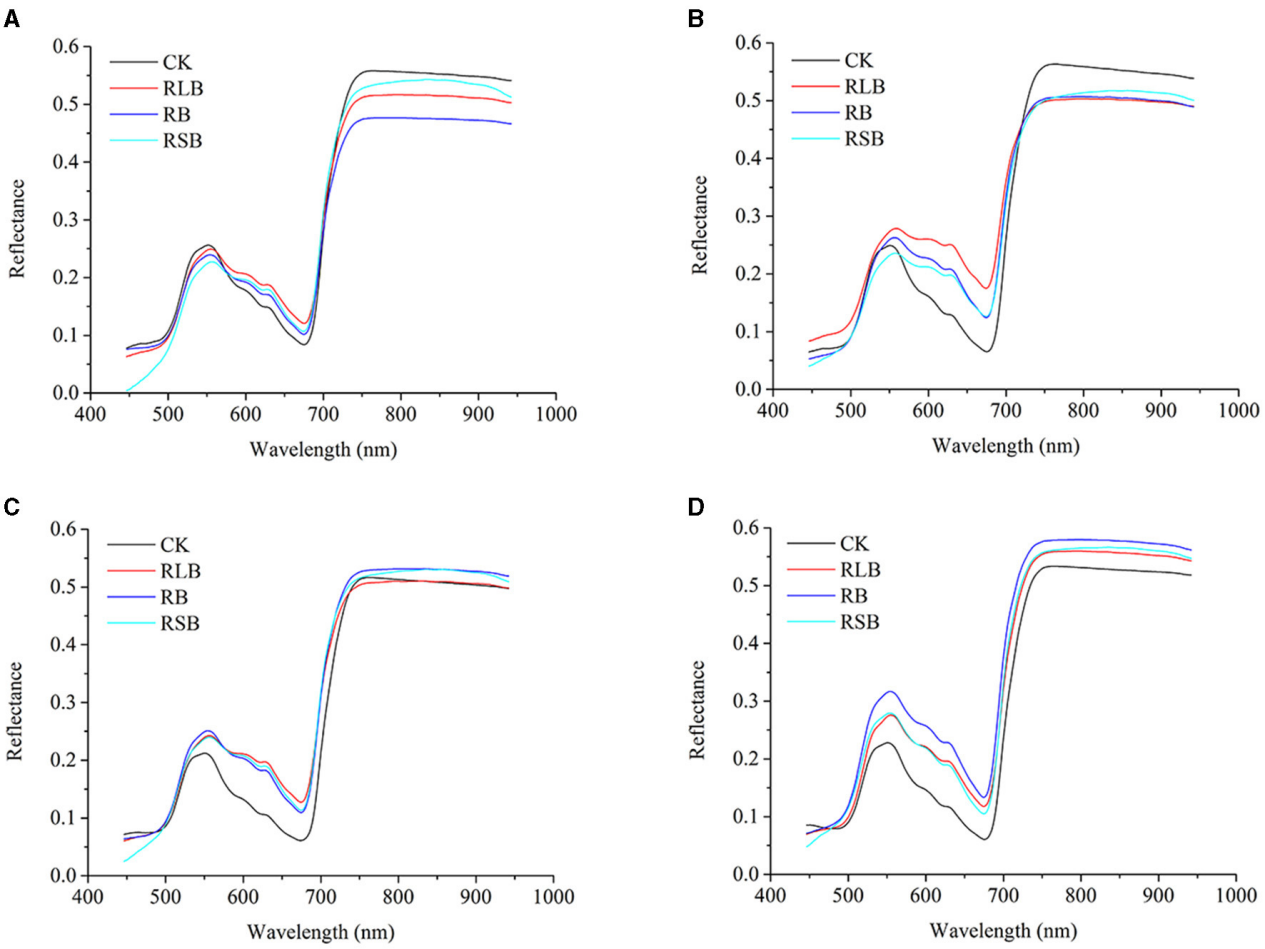
**(A–D)** represent the reflectance of rice varieties 01 (Zhongzheyou1), 02 (Jiuyou418), 03 (Zhongzao39), and 04 (Xiushui134).

To illustrate the difference among rice plants under different disease stress conditions, the average original spectra were plotted for visualization. In Figures 5A–D present healthy and disease-stressed leaves of the four rice cultivars, corresponding to rice varieties 01, 02, 03, and 04, respectively. The change tendency of these four varieties was similar to the spectral profile of the other two varieties of rice in the previous study (Feng et al., 2020).

The change tendencies of the rice leaves under different disease stress conditions were similar. The distinct difference could be observed in some ranges of wavelengths, including the range from 450 to 500, 580 to 680, and 720 to 940 nm. The difference among each class is a foundation for developing a classifier for rice disease detection. Besides, the distinction among different cultivars suggested that a discrepancy exists among different cultivars of rice under the same disease stress conditions. To perform a quantitative analysis for disease detection in rice, further processing should be applied to the spectra.

### Classification Models on Each Variety of Rice

The common CNN architecture was trained on four varieties of rice. The classification results are shown in Table 2. All the CNN models obtained an accuracy of over 97% for the training set and an accuracy of over 93% and over 87% for the validation set and the test set, respectively. These results indicated that the self-designed CNN was capable of extracting the features for discrimination of rice under different disease stress conditions. This provides the possibility for transferring the learned knowledge across different varieties.

**Table 2:** Classification results of the four varieties of rice.

**Variety**	**Tr^**a**^(%)**	**Val^**b**^(%)**	**Te^**c**^(%)**
01	100	93.75	100
02	97.08	94.12	87.5
03	100	94.74	94.44
04	100	100	94.74

^a^*Tr is the training set; ^b^Val is the validation set; ^c^Te is the test set*.

### Results With and Without Fine-Tuning

To verify the effectiveness of fine-tuning, the pre-trained CNN based on one rice variety was directly used to predict another rice variety without fine-tuning. The results of the prediction without fine-tuning are shown in Table 3.

**Table 3:** Classification results without and with fine-tuning.

**S-T^**a**^**	**Non-fine-tuning**			**Fine-tuning**			**Finetuning with a smaller set** ^**e**^		
	**Tr^**b**^(%)**	**Val^**c**^(%)**	**Te^**d**^(%)**	**Tr(%)**	**Val(%)**	**Te(%)**	**Tr(%)**	**Val(%)**	**Te(%)**
01–02	60.23	52.94	68.75	98.32	**88.24**	**87.50**	96.25	**88.24**	**87.50**
01–03	76.44	84.21	88.89	100.00	**89.47**	**88.89**	95.00	**89.47**	**88.89**
01–04	52.04	73.68	57.89	92.35	**89.47**	**89.47**	91.25	**89.47**	**89.47**
02–01	62.03	56.25	66.67	99.36	**93.75**	**86.66**	96.25	**93.75**	**86.67**
02–03	67.02	89.47	61.11	91.62	**89.47**	**88.89**	100.00	89.43	83.33
02–04	53.57	42.11	52.63	96.43	**94.74**	**89.47**	91.25	89.48	78.95
03–01	70.89	75.00	60.00	94.30	**93.75**	**93.33**	95.00	**93.75**	**93.34**
03–02	63.74	47.06	50.00	98.83	**88.24**	**87.50**	95.00	**88.23**	**87.50**
03–04	58.67	78.95	52.63	97.96	**100.00**	**89.47**	100.00	94.74	84.21
04–01	51.27	50.00	53.33	97.47	**93.75**	**86.67**	98.75	**87.50**	**86.67**
04–02	43.27	47.06	37.50	100.00	**88.24**	**87.50**	98.75	**88.24**	**87.50**
04–03	72.77	57.89	77.78	97.91	**100.00**	**88.89**	100.00	**94.74**	**88.89**

*^a^S-T means the source domain-the target domain; ^b^Tr means the training set of the target domain; ^c^Val means the validation set of the target domain; ^d^Te means the test set of the target domain; ^e^a small set only consisted of 20 samples of each class; the test set remained the same as in Table 1. The bold values means the accuracy rate of the test set was beyond 86%*.

Without fine-tuning, when 01 was the source domain, the accuracy of the test set of the target domains was lower than 69%, except for 03. When the CNN based on 02 was used to predict 04, it only obtained an accuracy of no more than 52% on both the training set and the test set. When 02 was transferred to 01 and 03, it also obtained unsatisfactory classification results. Besides, deterioration in classification results also occurred when CNNs trained on 03 were directly applied to predict 01, 02, and 04. This situation also happened when the source domain was 04.

After fine-tuning, the accuracy of the training set increased to more than 92%, while the accuracy of the validation set and the test set, respectively, increased to over 88 and 86%, with 10 out of the 12 transfer tasks obtaining an accuracy of over 87% on the test set. Compared with the results of the prediction without fine-tuning, the accuracy of the training set improved by 23–56.73%, while that of the test set improved by 18% on 10 out of the 12 transfer tasks. Besides, the accuracy after fine-tuning was close to the results of the CNN trained on each variety of rice, which suggested that fine-tuning was capable of transferring the feature representations learned from one variety of rice to another.

To further verify the effectiveness of fine-tuning, we also conducted fine-tuning with a smaller dataset of the target domain. The smaller training set only consisted of 20 samples of each class, and the validation set and the test set were kept the same. The training process was the same. As shown in Table 3, with a smaller dataset, the accuracy of the test set on nine out of all the transfer tasks is equal to or higher than that with the bigger dataset. Even though the accuracy of the test set of three transfer tasks declined, the amplitude of decline did not exceed 5.56%. The results indicated that fine-tuning could transfer the knowledge among different domains and that fine-tuning obtained satisfying results with a relatively small training set in the target domain.

### Results With Deep CORAL and DDC

The results of transfer learning with deep CORAL are listed in Table 4. Eight transfer tasks have achieved an accuracy of over 75% on the test set of the target domain. Compared with the results of non-fine-tuning, the accuracy for the test set of the target domain obtained improvements of over 15% on 8 out of the 12 transfer tasks. Furthermore, five of all the transfer tasks obtained an improvement of over 25%. It indicated that CORAL loss contributed to learning features that work well on the target domain. As for transfer task 03 → 01, the accuracy of the validation set and the test set of the target domain was 93.75 and 86.67%, respectively, which was slightly lower than the accuracy (93.33%) of the fine-tuning method.

**Table 4:** Classification results of deep CORrelation ALignment (CORAL) and deep domain confusion (DDC) (*fc*6^a^).

**S-T^**b**^**	**Deep CORAL**						**DDC**					
	**S** ^**c**^			**T** ^**d**^			**S**			**T**		
	**Tr^**e**^(%)**	**Val^**f**^(%)**	**Te^**g**^(%)**	**Tr(%)**	**Val(%)**	**Te(%)**	**Tr(%)**	**Val(%)**	**Te(%)**	**Tr(%)**	**Val(%)**	**Te(%)**
01–02	100.00	87.50	80.00	61.99	58.82	62.50	100.00	87.50	86.67	56.14	58.82	56.25
01–03	100.00	87.50	86.67	80.63	**84.21**	**83.33**	100.00	87.50	86.67	80.10	**84.21**	**83.33**
01–04	100.00	87.50	86.67	69.90	73.68	73.68	100.00	87.50	80.00	69.90	73.68	73.68
02–01	100.00	82.35	75.00	79.11	87.50	73.33	100.00	82.35	75.00	74.05	**81.25**	**80.00**
02–03	100.00	76.47	75.00	79.58	**78.95**	**77.78**	100.00	76.47	75.00	75.39	78.95	72.22
02–04	100.00	82.35	81.25	68.88	73.68	68.42	100.00	88.24	75.00	72.96	73.68	52.63
03–01	100.00	94.74	83.33	86.08	**87.50**	**86.67**	100.00	89.47	72.22	82.28	87.50	73.33
03–02	100.00	94.74	88.89	77.78	**76.47**	**75.00**	100.00	89.47	77.78	76.61	70.59	62.50
03–04	100.00	94.74	88.89	75.51	**84.21**	**84.21**	100.00	84.21	83.33	71.43	78.95	73.68
04–01	100.00	94.74	94.74	77.22	**81.25**	**80.00**	100.00	94.74	94.74	72.78	68.75	73.33
04–02	100.00	94.74	94.74	73.68	**76.47**	**75.00**	100.00	84.21	89.47	69.59	70.59	68.75
04–03	100.00	89.47	100.00	82.72	**84.21**	**77.78**	100.00	84.21	94.74	78.01	78.95	**77.78**

*^a^fc6 means that the last fully connected layer of the CNN was used for calculating domain loss (CORAL loss and maximum mean discrepancy, MMD, loss, as shown in Figures 3 and 4); ^b^S-T means the source domain-the target domain; ^c^S means the source domain; ^d^T means the target domain; ^e^Tr means the training set of the target domain; ^f^Val means the validation set of the target domain; ^g^Te means the test set of the target domain. The bold values means the accuracy rate of the test set was beyond 75%*.

Regarding DDC, the overall performance was inferior to the performance of deep CORAL. However, compared with the results with no fine-tuning, the accuracy for the test set of the target domain was improved by a rate over 10% on 8 out of 12 transfer tasks. Therein, three of the transfer tasks obtained an improvement of over 30%. The improvements suggested that MMD loss was of help in reducing the distance between the source domain and the target domain.

To investigate the choices of the layer, the *fc5* layer was adapted to compute domain loss in both deep CORAL and DDC. The results are listed in Table 5. Regarding deep CORAL, 4 out of all the 12 transfer tasks obtained an accuracy of over 75% on the test set of the target domain. In general, the accuracy of the target domain of each transfer task was slightly lower than their corresponding accuracy with the *fc6* as the transfer layer. In terms of DDC, six transfer tasks obtained an accuracy of over 73% on the test set of the target domain, while there was the case for seven transfer tasks with the *fc6* as the transfer layer. Overall, the *fc6* layer was more conducive for transferring the knowledge learned from one cultivar of rice to another for rice disease detection.

**Table 5:** Classification results of Deep CORAL and DDC (*fc*5^a^).

	**Deep CORAL**						**DDC**					
**S-T^**b**^**	**S** ^**c**^			**T** ^**d**^			**S**			**T**		
	**Tr^**e**^(%)**	**Val^**f**^(%)**	**Te^**g**^(%)**	**Tr(%)**	**Val(%)**	**Te(%)**	**Tr(%)**	**Val(%)**	**Te(%)**	**Tr(%)**	**Val(%)**	**Te(%)**
01–02	100.00	87.50	80.00	58.48	58.24	56.25	100.00	87.50	86.67	60.23	58.82	56.25
01–03	100.00	93.75	86.67	80.63	**84.21**	**83.33**	100.00	87.50	86.67	80.10	**84.21**	**83.33**
01–04	100.00	87.50	93.34	65.82	**73.68**	**73.68**	100.00	81.25	86.67	70.41	**73.68**	**73.68**
02–01	100.00	82.35	75.00	75.95	**81.25**	**73.33**	92.40	82.35	68.75	71.52	**75.00**	**73.33**
02–03	100.00	76.47	75.00	76.44	**77.78**	**78.95**	100.00	76.47	75.00	78.53	73.68	72.22
02–04	100.00	82.35	75.00	72.45	68.42	68.42	95.32	82.35	75.00	71.43	73.68	57.89
03–01	100.00	89.47	83.33	82.91	**87.50**	**80.00**	100.00	89.47	77.78	82.91	**87.50**	**73.33**
03–02	100.00	89.47	83.33	74.85	70.59	68.75	100.00	94.74	83.33	73.68	76.47	62.50
03–04	100.00	94.74	88.89	84.69	**89.47**	**73.68**	100.00	94.74	88.89	73.98	**78.95**	**73.68**
04–01	100.00	89.47	94.74	71.52	73.33	68.75	100.00	100.00	94.74	69.62	68.75	66.67
04–02	100.00	94.74	94.74	74.85	68.75	58.82	100.00	84.22	94.74	66.08	64.71	62.50
04–03	100.00	89.47	89.47	83.77	**84.21**	**77.78**	100.00	100.00	89.47	82.20	**78.95**	**77.78**

*^a^fc5 means that the penultimate fully connected layer of the convolutional neural network (CNN) was used for calculating domain loss (CORAL loss and MMD loss, as shown in Figures 3 and 4); ^b^S-T means the source domain-the target domain; ^c^S means the source domain; ^d^T means the target domain; ^e^Tr means the training set of the target domain; ^f^Val means the validation set of the target domain; ^g^Te means the test set of the target domain. The bold values means the accuracy rate of the test set was beyond 73%*.

Furthermore, the saliency map was applied to visualize the most informative wavelengths captured by the CNN model.

Saliency mapping is a technique for visualizing class models, and it is widely studied in computer vision. The method will numerically generate an image according to a learned classification network and a class of interest. The generated image is representative of the class in terms of the class scoring model (Simonyan et al., 2013). In computer vision, the magnitude of the class score defines the importance of the corresponding pixels for the class (Simonyan et al., 2013). We introduced this method into hyperspectral imaging analysis and the visualization of key wavelengths captured by a learned classification model. The interpretation of computing the class saliency using the class score derivative is that the magnitude of the derivative indicates which wavelength needs to be changed the least to affect the class score the most. To realize the visualization of the critical wavelength range, the class score was first computed by calculating the derivative (gradient) of the correctly classified class. Next, the wavelengths of each correctly classified sample were sorted by the absolute value of the corresponding gradient in descending order. Finally, the first 50 critical wavelengths of each sample of the same class were selected and counted according to the frequency of wavelengths. After obtaining the critical wavelengths and their corresponding frequencies, the wavelength saliency map was plotted for visualization. Here, the results of 01 were used for visualization. Since there were only 15 samples of the test set of 01, there were too few to visualize the critical wavelengths. The validation set and the test set of 01 were concatenated (called the combined set) and used for prediction with the CNN model in Table 2 and the deep CORAL in Table 4. Then, the results were used for the visualization of the critical wavelength range. On the one hand, the combined set results of the CNN model built on the data of 01 alone (the prediction accuracy of the combined set was 96.77%) were used to visualize the critical wavelength range without transfer learning. On the other hand, the combined set results of 01 after achieving the transfer task 03 → 01 (the prediction accuracy of the combined set was 90.32%) with deep CORAL were used to visualize the key wavelengths after transfer learning.

**Figure 6:**
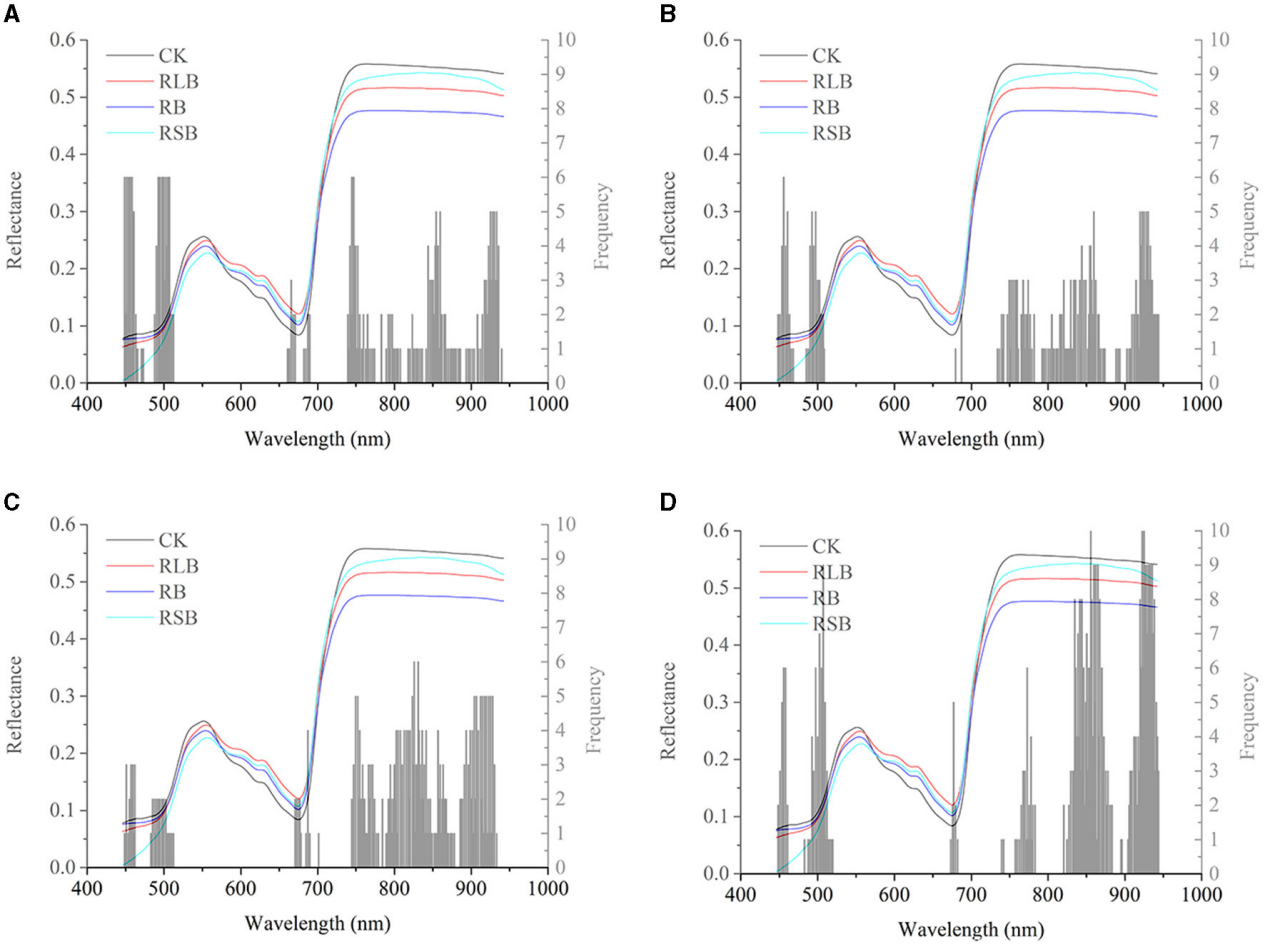
Saliency map before transfer learning regarding the rice variety 01 (Zhongzheyou1). **(A–D)** represent the key wavelength ranges captured by CNN to correctly classify rice diseases with classes 0 (healthy samples, CK), 1 (rice leaf blight, RLB), 2 (rice blast, RB), and 3 (rice sheath blight, RSB), respectively.

Figure 6 shows the differences and connections between the spectral profile and the saliency map. In Figures 6A–D represent the frequencies of the critical wavelengths of classes 0, 1, 2, and 3, respectively. Without transfer learning, the wavelengths of interest are located in almost the same ranges, including the range from 448 to 467 nm, the range from 485 to 510 nm, the range from 660 to 690 nm, and the range from 740 to 940 nm. Compared with the wavelength range discussed in section Spectral profile spectral profile, there were several common wavelength ranges, including the range from 448 to 467 nm, the range from 485 to 510 nm, and the range from 740 to 940 nm. It indicated that the result of the saliency map was largely consistent with the spectral profile.

**Figure 7:**
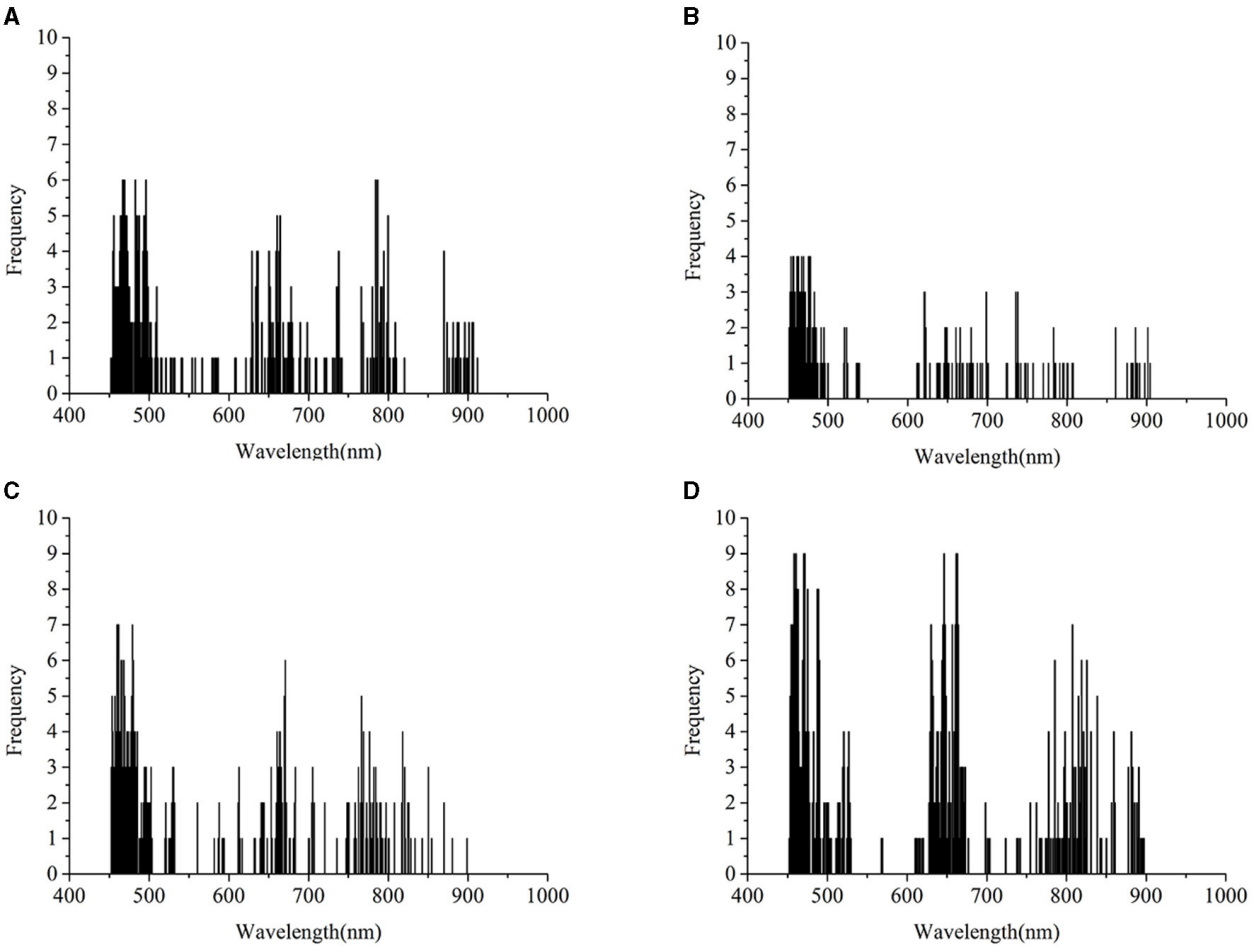
Saliency map after transfer learning regarding the rice variety 01 (Zhongzheyou1), which is based on the results of 01 after achieving the transfer task 03→ 01 with deep CORAL. **(A–D)** Represent the key wavelength ranges captured by CNN to correctly classify rice diseases with classes 0 (healthy samples, CK), 1 (rice leaf blight, RLB), 2 (rice blast, RB), and 3 (rice sheath blight, RSB), respectively.

In Figure 7, the wavelengths after transfer learning were located in a similar range as they were without transfer learning, including the ranges from 450 to 500 nm, the range from 518 to 525 nm, the range from 535 to 540 nm, the range from 600 to 750 nm, and the range from 760 to 910 nm. Overall, the range of feature wavelengths captured by the CNN with transfer learning had a significant overlap with the wavelength range captured by the CNN without transfer learning.

The intersection of the key wavelengths with and without transfer learning suggested that deep CORAL was able to learn the features that work well on both the source domain and the target domain. Furthermore, CORAL loss, as a part of a total loss, contributed to the reduction in the distance between the source domain and the target domain. Transfer learning also had the capability to learn knowledge that could be applied to a different domain.

## Discussion

Transfer learning is able to address the problem that a predictive model based on specific data cannot work well on another data source from a related domain but under different distribution conditions. According to the saliency map shown in Figures 6, 7, it appears that the critical wavelength range is similar in cases with and without transfer learning. In this study, three different deep transfer learning methods were applied for disease detection among different cultivars of rice. The results showed that deep transfer learning methods could perform disease detection across different rice cultivars in an efficient manner. Among the methods used in this study, fine-tuning was an easily operative and effective transfer learning method, obtaining the best overall performance after transfer learning. It was easy to operate and obtain the accuracy for the target domain, which was equal to or just slightly lower than the accuracy of the CNN directly built on the corresponding target domain. Besides, deep CORAL, as a feature-based transfer learning method, has the ability to learn the feature representation that works well on both the source domain and the target domain. Overall, compared with the result of DDC, deep CORAL performed better. In the study of Sun et al. (2016), the CORAL-based method was also shown to be superior to the MMD-based method. The reason could be that CORAL, as an asymmetric transformation, tries to “bridge” the two domains, while MMD as a symmetric transformation tries to find a space that ignores the difference between the source and the target domain (Sun et al., 2016). Although deep CORAL has achieved good predictions on both the source domain and the target domain among most of the transfer tasks, the accuracy was not as good as the results of direct modeling and fine-tuning. This situation also existed in other studies. Some traditional transfer learning methods obtained an average accuracy lower than 60% on the standard Office dataset, such as geodesic flow kernel (GFK) (Gong et al., 2012) and subspace alignment (SA) (Fernando et al., 2013). The CORAL method obtained an accuracy lower than 65% on four of the transfer tasks and an average of 69.4% for all the six transfer tasks with the same dataset (Sun et al., 2016). Furthermore, deep CORAL obtained an average accuracy of 72.1% on the same dataset. Long et al. improved the average accuracy to 72.9% on the same dataset with deep adaption networks (Mingsheng et al., 2015).

To achieve better performance with transfer learning, a deep network should be carefully designed. The deep network should obtain good predictions based on the data of the source domain. Thus, the deep network allows the extraction of feature representations that are conducive to achieving classification. Given that the resnet architecture is one of the most excellent deep neural network architectures, deep transfer learning based on resnet was carried out for a comparison with the results based on the customized CNN architecture. To avoid overfitting, ResNet14 (the architecture is provided in Supplementary Table 1) was used for transfer learning, and the global average pooling layer was set as the transfer layer. The results based on ResNet14 are provided in Supplementary Table 2. Comparing with the results shown in Table 4, the results based on ResNet14 were similar or inferior to the results based on the customized CNN. Therefore, a well-designed CNN architecture was a prerequisite for a good transfer effect. In this case, since the spectral data were one-dimensional, a relatively shallow CNN architecture was suitable for the classification task.

In addition, the pair-wise transfer was widely studied in the field of computer vision, and it was shown to be effective for the classification of rice diseases in the above sections. Moreover, it was worth investigating whether a multi-task transfer strategy could achieve better performance. The multi-task transfer strategy could potentially improve the transfer performance for this strategy by increasing and enriching the training data. Considering the large number of transfer tasks, only the case of joint training of three rice transfers to one rice variety with deep CORAL was explored. Thus, the multi-task transfer strategy was implemented based on the self-designed CNN in section Material and Methods with *fc*6 as the transfer layer, which first jointly trained on three rice varieties (e.g., 02, 03, and 04) and then transferred to the fourth one (e.g., variety 01). The results of the multi-task transfer are provided in Supplementary Table 3. With the deep CORAL method, the accuracy of the test set of the target domain was improved from 86.67 to 93.33% compared with the result (03–01) shown in Table 4. In addition, the accuracy of the test set of the target domain was improved from 83.33 to 88.89% compared with the result (01–03) of deep CORAL shown in Table 4. A similar improvement occurred when the target domain was 02 or 04. Therefore, the multi-task transfer strategy could contribute to obtaining better performance, which alleviated the challenge of transfer learning with a limited amount of data. Furthermore, it is worth studying which varieties are selected and how they are selected for joint transfer when there are a large number of varieties of rice. Besides, it contributes to confusing domains by adding domain loss both in deep CORAL and DDC. According to some related studies (Tzeng et al., 2014; Sun and Saenko, 2016; Sun et al., 2016), the last few fully connected layers were usually applied for calculating domain loss. In this study, the choices for the layer for calculating domain loss were studied. The results showed that there was a difference in using a different layer for calculating domain loss. Therefore, this observation should be taken into account in transfer learning. Generally, CORAL and MMD are kernel tricks that try to measure the distance between the source domain and the target domain in another space. To improve the transferability of transfer learning in spectral analyses, some kernel tricks could be introduced and combined into the spectral data metric. In addition, Long et al. proposed a multiple kernel variant of MMD to realize transfer learning, which combined with the last three fully connected layers for domain loss. This method was beyond discussion in this study and could be investigated in future studies. Considering the balance of classification loss and domain loss, the hyperparameter λ played a key role in this aspect, which also causes difficulty for training the network in transfer learning to some extent. Some previous studies on computer vision have tended to set it to 1 according to previous experience (Mingsheng et al., 2015; Sun and Saenko, 2016), while some set λ to 0.25 (Tzeng et al., 2014). According to the experience of the authors, the setting of λ to 0.01 was better at the first stage of training, and then it was expanded to 0.1 and 1.

Considering practical application, among the previous studies that focused on rice disease detection with a spectroscopy technique, it was hard to find a network trained on a specific variety that could be directly applied to another variety. However, it will cost much time and energy to build and train a deep network for each cultivar of rice. Transfer learning is a promising tool for solving this problem. Transfer learning can learn feature representations that work well on different but related tasks. Since deep learning is good at automatically extracting features of different levels, it is quite desirable for combining deep learning networks and transfer learning together to realize transferability across different tasks. This study showed the feasibility of combining deep transfer learning with spectra data for rice disease detection. In future studies, more samples need to be used to further improve performance. If a high-quality spectral database like ImageNet could be established and maintained, it will strongly promote the development of transfer learning with spectral analyses and contribute to the development of practical applications.

## Conclusions

In this study, hyperspectral imaging was performed to acquire the information of four cultivars of rice under different disease stress conditions. Deep transfer learning was introduced for the first time to rice disease detection across different rice cultivars simultaneously. A self-designed CNN architecture was developed as a classification model and basic network of deep transfer learning. The transfer learning methods used in this study were fine-tuning, deep CORAL, and DDC. The results illustrated that the fine-tuning method was a relatively easy and efficient solution for rice disease detection across different rice cultivars. Deep CORAL was capable of transferring the knowledge learned from a specific variety to another variety and was superior to DDC in overall performance. In addition, when jointly training on three varieties of rice and then transferring to the fourth one, the accuracy of the target domains improved. This indicated that the multi-task transfer strategy could improve transfer performance, which increased and enriched the training data.

Nevertheless, there existed limitations in this study. Rice leaves were collected and explored from only four different cultivars. Rice samples of more cultivars were suggested to be collected to study the universality of deep transfer learning methods. The number of samples of each cultivar was no more than 250. The small size of a dataset may restrict the performance of deep learning methods and transfer learning combined with deep learning. More samples may lead to the better performance of deep transfer learning methods. The results of this study showed that it is feasible to combine spectral data with deep transfer learning for the classification of rice diseases and that the inclusion of more samples and the use of emerging transfer learning methods are worth further study. A relevant standard database of spectral data, like ImageNet in the field of computer vision, with different rice diseases could be organized and developed, which would be a valuable resource for researchers, educators, and students.

In future studies, transfer learning methods can be extended to more scenarios, such as different regions and different equipment. Transfer learning has great potential for rice disease detection and will contribute to the translation of relevant researches into practical applications in an efficient manner.

## Data Availability Statement

The raw data supporting the conclusions of this article will be made available by the authors, without undue reservation.

## Author Contributions

LF and CZ: conceptualization, formal analysis, and supervision. CZ and BW: data curation and validation. YH, LF, and CZ: funding acquisition and writing, project administration review and editing. CZ: investigation, methodology, and software. CZ and YH: resources. BW and LF: visualization and writing, original draft.

## Funding

This study was supported by XPCC Science and Technology Projects of Key Areas (2020AB005) and the National Natural Science Foundation of China (31871526, 61705195).

## Conflict of Interest

The authors declare that the research was conducted in the absence of any commercial or financial relationships that could be construed as a potential conflict of interest.

## Publisher's Note

All claims expressed in this article are solely those of the authors and do not necessarily represent those of their affiliated organizations, or those of the publisher, the editors and the reviewers. Any product that may be evaluated in this article, or claim that may be made by its manufacturer, is not guaranteed or endorsed by the publisher.
