# Supplementary material for: Hyperspectral Imaging Combined With Deep Transfer Learning for Rice Disease Detection
Source: Front Plant Sci. 2021 Sep 29;12:693521. doi: 10.3389/fpls.2021.693521 (PMC8511421; doi:10.3389/fpls.2021.693521)
Supplement: Supplementary file 1 [file Data_Sheet_1.docx]

Supplementary Material

# Supplementary Figures and Tables

## Supplementary Figures

| 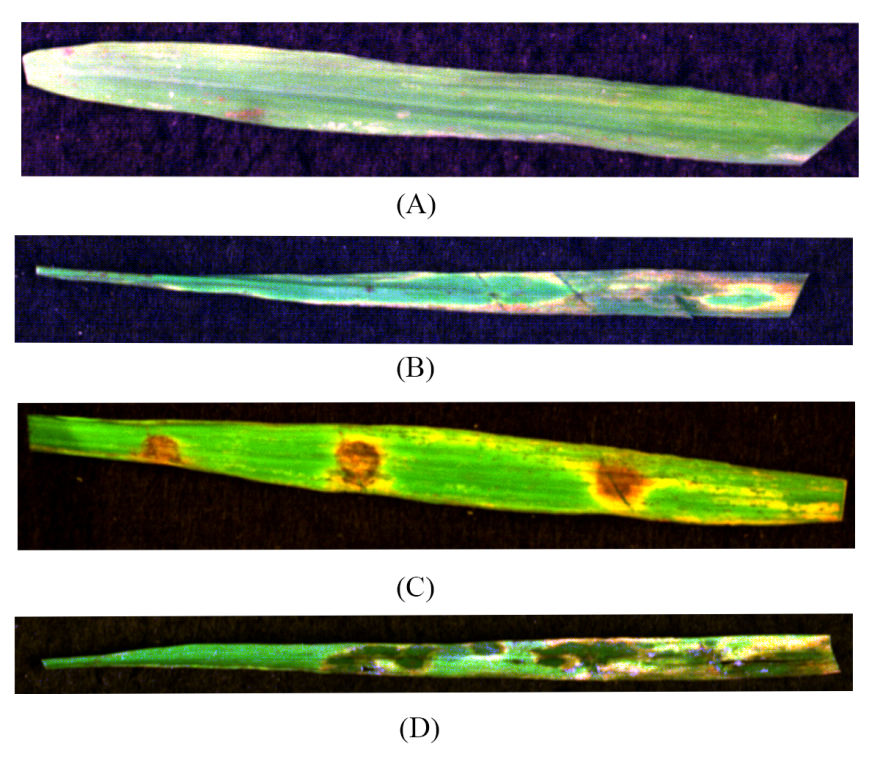 |
| --- |

**Supplementary Figure 1.** (A) CK of Zhongzao39 (healthy sample); (B) RLB of Zhongzao39 (disease sample); (C) RB of Zhongzao39 (disease sample); (D) RSB of Zhongzao39 (disease sample).

| **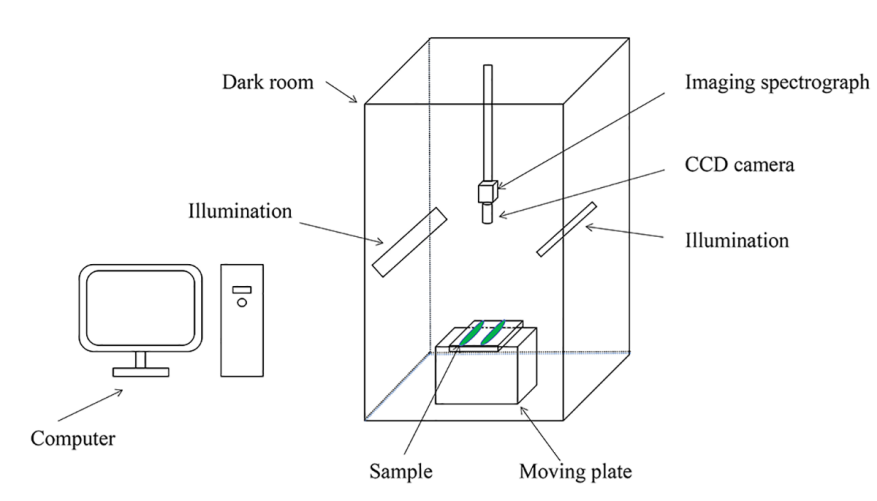** |
| --- |

**Supplementary Figure 2.** Collection platform

| 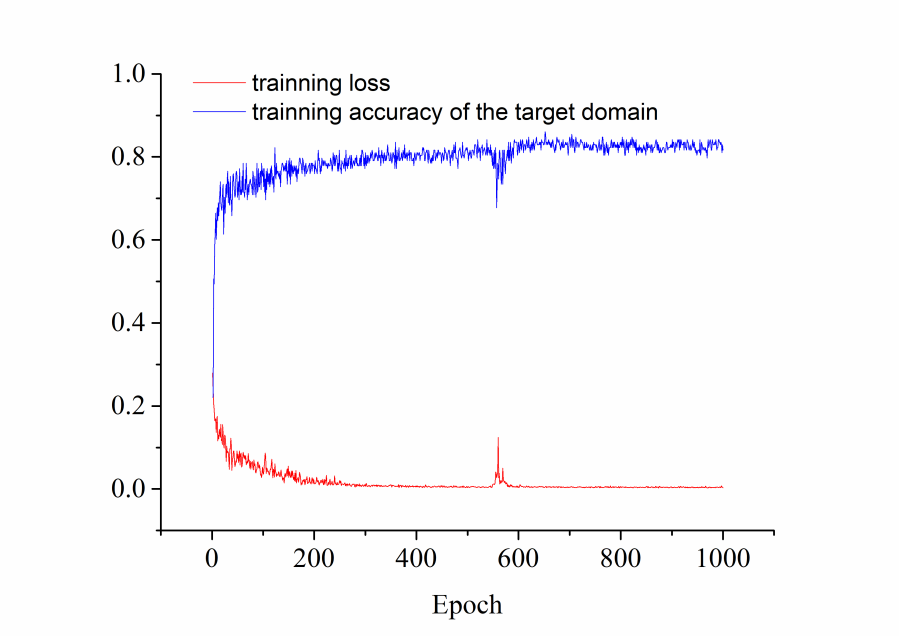  (A) | 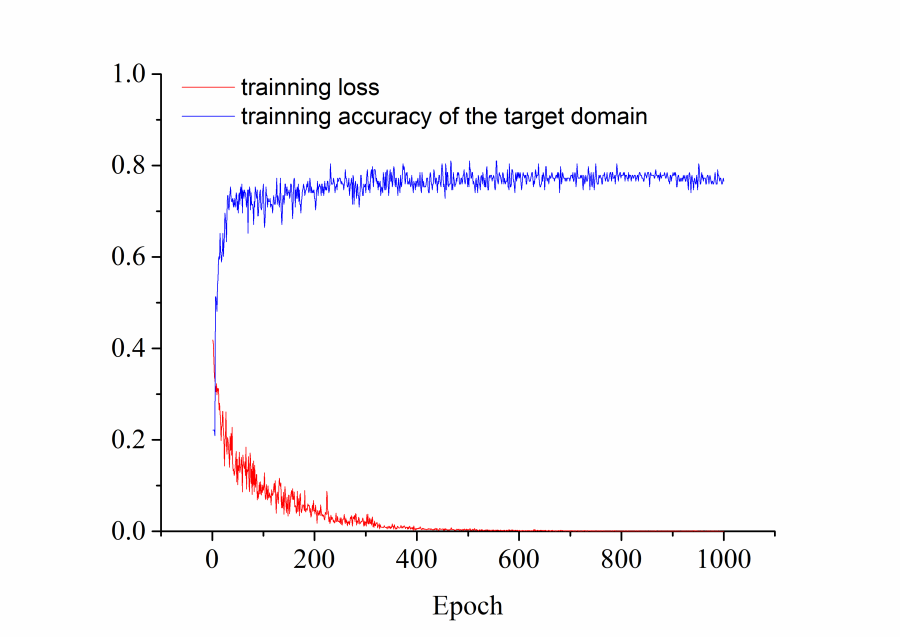  (B) |
| --- | --- |

**Supplementary Figure 3.** (A) The relationship between epochs and training performances of the target domain with Deep CORAL transfer learning method; (B) The relationship between epochs and training performances of the target domain with DDC transfer learning method.

## Supplementary Tables

**Supplementary Table 1** The architecture for ResNet14. Building blocks are shown in brackets with the number of blocks stacked.

| **Layer name** | **14-layer** |
| --- | --- |
| Conv1 | 7×7, 128, stride 2, padding 3 |
| Conv2_x | 3×3 max pool, stride2, padding 1 |
|  | 3×3, 128  ×2  3×3, 128 |
| Conv3_x | 3×3, 64  ×2  3×3, 64 |
| Conv4_x | 3×3, 16  ×2  3×3, 16 |
| Fully-connected layer | average pool, 4-d fc, softmax |

**Supplementary Table 2** The classification results of Deep CORAL based on ResNet14 (*fc*6^a^)

| S-T^b^ |  |  | **Deep CORAL** | | | |
| --- | --- | --- | --- | --- | --- | --- |
|  | S^c^ | | | T^d^ | | |
|  | Tr^e^ | Val^f^ | Te^g^ | Tr | Val | Te |
| 01-02 | 96.84% | 75.00% | 80.00% | 80.00% | 67.25% | 64.71% |
| 01-03 | 96.84% | 87.50% | 86.67% | 78.53% | **89.47%** | **88.89%** |
| 01-04 | 98.10% | 87.50% | 80.00% | 63.27% | 73.68% | 73.68% |
| 02-01 | 99.42% | 76.47% | 75.00% | 70.89% | 81.25% | 73.33% |
| 02-03 | 100.00% | 82.35% | 85.00% | 78.53% | 78.95% | 72.22% |
| 02-04 | 95.91% | 76.47% | 81.25% | 62.76% | 68.42% | 63.16% |
| 03-01 | 100.00% | 84.21% | 83.33% | 87.34% | **93.75%** | **80.00%** |
| 03-02 | 100.00% | 84.21% | 77.78% | 76.02% | **76.47%** | **75.00%** |
| 03-04 | 100.00% | 84.21% | 77.78% | 73.98% | **84.21%** | **78.95%** |
| 04-01 | 95.92% | 84.21% | 89.47% | 70.89% | 75.00% | 73.33% |
| 04-02 | 94.39% | 89.47% | 89.47% | 68.42% | 70.59% | 62.50% |
| 04-03 | 96.43% | 100.00% | 89.47% | 75.39% | **89.47%** | **77.78%** |

^a^ *fc6* means the last fully connected layer of the CNN was used for calculating domain loss (CORAL loss and MMD loss, as shown in Fig. 2.and Fig.3.); ^b^ S-T means the source domain-the target domain;^c^ S means the target domain; ^d^ T means the target domain; ^e^ Tr means the training set of the target domain; ^f^ Val means the validation set of the target domain; ^g^ Te means the test set of the target domain.

**Supplementary Table 3** The classification results of multi-task transfer with Deep CORAL (*fc*6^a^)

| S-T^b^ |  |  | **Deep CORAL** | | | |
| --- | --- | --- | --- | --- | --- | --- |
|  | S^c^ | | | T^d^ | | |
|  | Tr^e^ | Val^f^ | Te^g^ | Tr | Val | Te |
| (02+03+04)-01 | 99.82% | 89.09% | 81.25% | 86.71% | **93.75%** | **93.33%** |
| (02+03+04)-02 | 98.35% | 98.15% | 88.46% | 81.87% | **86.11%** | **81.25%** |
| (01+02+04)-03 | 99.62% | 85.92% | 84.00% | 86.39% | **94.74%** | **88.89%** |
| (01+02+03)-04 | 100.00% | 84.62% | 87.75% | 77.04% | **89.47%** | **84.21%** |

^a^ S-T means the source domain-the target domain, e.g., first jointly training on three rice varieties (02, 03 and 04) and then transferring to the fourth one (01);^b^ S means the target domain; ^c^ T means the target domain; ^d^ Tr means the training set of the target domain; ^e^ Val means the validation set of the target domain; ^f^ Te means the test set of the target domain.
